# Successful 3D culture and transplantation of mouse isolated preantral follicles in hydrogel of bioengineered Wharton’s jelly

**DOI:** 10.1371/journal.pone.0290095

**Published:** 2023-09-20

**Authors:** Elnaz Zand, Elias Rajablou, Seyedeh Fatemeh Siadat, Bahare Beiki, Vahid Akbarinejad, Christiani Andrade Amorim, Mojtaba Rezazadeh Valojerdi, Leila Alsadat Tahaei, Rouhollah Fathi

**Affiliations:** 1 Department of Embryology, Royan Institute for Reproductive Biomedicine, Reproductive Biomedicine Research Center, Academic Center for Education, Culture and Research (ACECR), Tehran, Iran; 2 Faculty of Veterinary Medicine, Garmsar Branch, Islamic Azad University, Garmsar, Iran; 3 Department of Biology, North Tehran Branch, Islamic Azad University, Tehran, Iran; 4 Skin and Stem Cell Research Center, Tehran University of Medical Science, Tehran, Iran; 5 Faculty of Veterinary Medicine, Department of Theriogenology, University of Tehran, Tehran, Iran; 6 Institut de Recherche Expérimentale et Clinique, Pôle de Recherche en Physiopathologie de la Reproduction, Université Catholique de Louvain, Brussels, Belgium; 7 Faculty of Medical Sciences, Department of Anatomy, Tarbiat Modares University, Tehran, Iran; Jinan University, CHINA

## Abstract

**Main objective:**

Due to Human Wharton’s Jelly (HWJ) could be applied in tissue engineering as a bio scaffold, the present study was conducted to investigate the effects of HWJ hydrogel on in vitro culture and auto-transplantation of mouse ovarian follicles.

**Materials and methods:**

HWJ was isolated from umbilical cord and decellularized with SDS/Tris/EDTA. DNA, Collagen and Glycosaminoglycans (GAGs) were measured. Decellularized Wharton’s Jelly (DWJ) was dissolved to make Wharton’s Jelly Hydrogel (WJH), and composited with Alginate (ALG) (1.5%) in equal ratio (WJH+ALG). Then, mouse preantral follicles were isolated and encapsulated in 10μL droplets of WJH and randomly considered for both 14 days culture and auto-transplantation.

**Results:**

Collagen, GAGs and DNA evaluations showed majority of WJ cells have been removed and MTT approved no toxicity. Degradation rate and rheological analysis represented optimal hydrogel compatibility. The data from in vitro culture revealed significant antral formation in WJH+ALG (P≤0.05). In transplantation, follicles failed to survive in ALG; however, survived in WJH+ALG to antral stage (P<0.05). VEGF and CD34 had greater expression in WJH+ALG than ALG (P< 0.05).

**Conclusion:**

Wharton’s jelly hydrogel and Alginate compound is interesting composite for successful development of mouse preantral follicles in both 3D in vitro culture and transplantation.

## Introduction

Subfertility is a serious concern in women afflicted with cancer and undergoing gonadotoxic medical therapies including chemotherapy and radiotherapy [1]. Premature subfertility, by itself, has a negative effect on the quality of life in these patients, and hence, fertility preservation plays a key role in this regard. There are quite a number of ways patented to preserve fertility in cancer patients like oocyte, embryo and ovarian tissue cryopreservation [2]. However, ovarian tissue cryopreservation, by itself, may result in its own restrictions while being applied for critical cancer conditions due to its contamination with malignant cells, which potentiates recurrence of cancer in the patient following ovarian tissue transplantation. That is why numerous studies have been conducted to develop alternative strategies including in vitro activation of primordial follicles, in vitro culture of preantral follicles and construction of transplantable artificial ovary in order to preempt the potential reintroduction of malignant cells to patient’s tissues and organs [2,3]. With the latter, preantral follicles are isolated from ovarian tissue and would be enclosed in a matrix so as to emulate the natural three-dimensional structure of ovary for isolated follicles. Indeed, reproductive medicine has paved a new path to utilize tissue engineering and regenerative medicine techniques to develop alternatives to restore fertility in cancer patients or other women suffering from any type of ovarian disorders [4]. As a result, the key factors are found to be biomaterials, playing crucial roles while replacing the natural environment of isolated cells or explanted tissue so that they can give assurance and maintain their three-dimension structure and provide the demanded biological and mechanical signals [5]. Natural components like collagen, fibrin, alginate and decellularized natural and synthetic tissues such as PEG and so forth are used in biomaterial applications [6–8]. In reproductive biomedicine, hydrogel plays a dominant role in fertility preservation procedure of those suffering from ovarian dysfunction. It also comes along with advantages like biocompatible, biodegradable, low immunogenicity and porous characteristics required/ demanded to allow diffusion of nutrients [9].

With respect to assembly of artificial ovary, an appropriate scaffold with optimum degradability to support follicular development, cell migration and angiogenesis is of utmost significance and various natural, and synthetic polymers have been applied for encapsulation of isolated follicles as well. In this regard, one of the scaffold materials that have been used for encapsulation of ovarian follicles is alginate, which is a family of polysaccharides composed of copolymers of b-D-mannuronic acid (M) and a-L-guluronic acid and is generated by brown algae and bacteria. While alginate is easy-to-handle, versatile, nonantigenic, biocompatible and biodegradable, it is an inert polymer without adhesion molecules, which may negatively influence cell survival and proliferation. Moreover, its slow degradation rate limits homogeneous vascularization throughout the matrix. To circumvent these problems, alginate can be combined with biological scaffolds consisting of extracellular matrix, as they are composed of a complex combination of macromolecules including glycosaminoglycans, collagen, laminin and fibronectin as well as a plethora of growth factors. Such features mimic microenvironment more efficiently that cell require for proliferation, differentiation, migration, homing, angiogenesis and normal function. In this context, there are successful reports on utilization of natural ECM for tissue engineering of heart, kidney, and intestine [5,10].

One of the natural tissues used in biomaterials is Wharton’s Jelly (WJ), a connecting tissue which has one vein and two arteries and is located within the umbilical cord (UC) and thus is a good source of nutrient and growth factors [11,12]. The ECM found in WJ is considered as a proper source because of its low cell content rate and rich collagen, Glycosaminoglycan (GAG), IGF-1, PDGF, EGF and TGF-β-1 amounts [13]. Wharton’s Jelly Hydrogel (WJH) is a suitable platform to promote intracellular communication, matrix cell-biomaterial interactions and secretion of signaling molecules [8]. However, WJH has not been used for culture of ovarian follicles or construction of artificial ovary to our knowledge. Therefore, the objective of this study was to evaluate the influence of WJH on in vitro 3D culture of isolated ovarian follicles and development of encapsulated follicles following auto-transplantation.

## Methodology (martials & methods)

The current study was performed based on Royan institute (Tehran, Iran) ethical committee guideline (Ethical committee approval NO: IR. ACECR. Royan. REC.1396.171) and performed in three steps:

1. Human Wharton’s jelly preparation, decellularization and hydrogel production
2. Mouse follicular 3D culture using human Wharton’s jelly hydrogel
3. Mouse follicular 3D transplantation using human Wharton’s jelly hydrogel

### Step 1: Human Wharton’s jelly preparation, decellularization and hydrogel production

**Fig 1:**
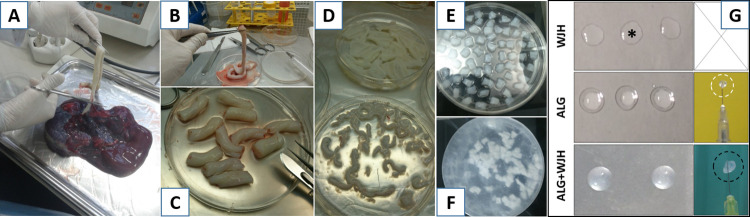
Different stages of WJ hydrogel (WJH) preparation. A) cutting umbilical cord from placenta, B) washing the umbilical cord, C) cutting umbilical cord to small fragments, D) removing the vessels, E) decellularization of Wharton’s jelly, F) Lyophilization of decellularized Wharton’s jelly and G) droplets of WJH and its combination with alginate (ALG+WJH) ready to 3D follicular culture and transplantation.

In this study, the 14 UCs tissues were collected from healthy women with no immunodeficiency virus (HIV), hepatitis virus type B, hepatitis virus type C, or syphilis, (ages between 30–35 years old) who had cesarean section in Arash Hospital (Tehran-Iran). The women included in the study were asked in advance to fill out informed consent form to authorize the experimental use of their tissues. Then plunged in Dulbecco’s phosphate- buffered saline (PBS, pH = 7.4; Gibco; UK) containing 1% streptomycin and penicillin and stored at 4°C. Following that, they were washed several times with PBS solution, containing 1% streptomycin, to remove the blood cells and debris. Through the next stage, the tissues were cut into 3–4 cm fragments, opened in the middle and peeled off the outer and vascular tissue to obtain WJ. It should be stated here that sterile conditions were observed throughout all the procedures as well (Fig 1).

Having finished WJ isolation procedures, decellularization protocol was performed as per the steps stated below:

The WJ was then plunged into hypotonic Tris- Buffered- Salin (TBS, 10 mM) with pH = 5 within ethylene diamine tetra acidic acid (EDTA 0.1% w/v) at 4°C for 16 h and was treated with a 0.03% sodium dodecyl sulfate (SDS) in TBS and EDTA, pH = 7.6 for 24 h at RT under shaking. Through the next step, the samples were washed in TBS to remove SDS. The final solution included Tris hydrochloric acid (50 mM) and Magnesium Chloride (10 mM), pH = 7.5 at 37 for 3 h. As the eventual stage, WJ tissues were washed with PBS for 24 h by agitation and lyophilization for subsequent experiment.

### 1–2 Assessments of the decellularized Wharton’s jelly

#### 1-2-1 Histological Analysis

Both indigenous and decellularized HWJ were analyzed with histology evaluation techniques. Each sample was fixed in Blouin’s and Formalin 10% for 24 h and 72 h respectively, and then embedded in paraffin wax and sectioned with 5 μm thickness. The tissue slides were processed using the standard staining procedures as follows: Hematoxylin and Eosin (H&E), Masson’s Trichrome (for Collagen). Applying 4, 6-diamidino-2-phenylindole (DAPI), each sample was labeled in order to clarify any DNA left in the tissue under fluorescent rays. All sections were observed under a light microscope (Olympus, Germany).

#### 1-2-2 DNA quantification, Glycosaminoglycan’s (GAGs) and Collagen content

DNA quantity applied both on intact and decellularized human Wharton’s Jelly (HWJ). Scaffolds was homogenized in lysis buffer (50 mM Tris- HCl, 50 mM EDTA, 1% SDS and 10 mM NaCl with pH = 8) and was located in a water bath of 10 λ proteinase K overnight at 55°C.This vortex solution was then put in RT to form a two-phase feature. Following that, 0.5 mL Phenol Chloroform was added to this solution and centrifuged for 20 minutes at 4°C (13700 rpm). The top part of this aqueous phase which has got DNA was transferred into a new tube to which an equal volume of Chloroform was added. Having this solution centrifuged, we came to two more phases out of which the upper part was removed and replaced with equal amount of ethanol 100% which was centrifuged for 15 minutes at 4°C (13700 rpm). Then the same procedures were performed for 70% ethanol. Later on, the derived pellet was dissolved in Tris-HCl for 15minutes at 37°C and the content of DNA was finally measured by the spectrophotometer at 260 nm (Thermo Fisher). To evaluate the total GAG content (μg/mg) in intact and decellularized HWJ per dry tissue weight, Blyscan assay (Biocolor life, science, UK) was used according to the manufacture datasheet. Finally, the GAGs concentration was measured at 565 nm. S2000 SIRCOL kit (Biocolor, UK) was applied to calculate the total Collagen content in WJ-ECM, according to the manufacturer’s instruction. The total Collagen concentration was performed by evaluating absorbance at 555 nm by micro plates spectrophotometer.

#### 1-2-3 Scanning electron microscopy (SEM)

To assess the ultrastructure of the scaffolds, morphological analysis was conducted taking advantage of SEM. In this regard, the samples were fixed in 2.5% glutaraldehyde for one day, and then were washed twice with PBS for 10 minutes and plunged into the 1% Osmium Tetroxide for 2 h. The samples were then dehydrated through an increased graded of ethanol at 30, 50, 70, 90 and 100% (20 minutes for each concentration). Following this stage, scaffolds were fixed onto polaren suptturn coater and were coated with thin layer gold at 10–24 KV. During the finally, the samples were observed under a KY-EM 3200 scanning electron microscopy (XL30) in Amirkabir University of technology (Tehran, Iran).

### 1–3 Preparation of Wharton’s jelly hydrogel

In order to make a hydrogel, WJ was first lyophilized. Then 30 mg of lyophilized WJ was weighted and dissolved in 1ml digest solution including acetic acid 0.5 M and 1mg/ml pepsin and then was magnetically stirred for at least 48 hours at RT. Through the coming stage, the solutions containing different densities of lyophilized WJ were neutralized with NaOH 2M and then got mixed with ALG (1.5%), was prepared by dissolving 0.15 g sodium alginate in 10 mL NaCl in ratio of 1:1 with WJH. To get it synchronized, it was relocated on stirrer machine for at least 3 hours. To form WJH+ALG a few droplets of CaCl2 were added as calcium bath.

#### 1-3-1 Metabolic activity and viability test (MTS assay)

The evaluation of MTS (3-(4, 5-dimethylthiazol-2-yl)-2,5 diphenyltetrozolium bromide) was performed to find out the toxicity rate of WJH. The cultured medium was exposed to the WJH bead for 72 h. Following that, 2×10^4^ cells of mouse embryonic fibroblast (MEF) were cultured in 96-well and were exposed to the conditioned medium of WJH for 24–48 and 72 h. This procedure was followed by adding MTS and incubated at 37°C for at least 3–4 h. The absorbance rate was then evaluated at a wave length 490 nm by Elisa reader (Thermo Scientific, Multiskan Spectrum).

#### 1-3-2 Rheological measurement and weight loss

Hydrogel mechanical properties of ALG and WJ were measured by storage modulus (*G*′) and loss modulus (*G*″). The hydrogel samples were prepared to yield discs of 2 mm diameter and 0.5 mm thickness by rheometer device (MCR 300) (Institute for color science and technology, Tehran, Iran).

In our study, primary weight loss of WJH was assessed. The hydrogels were soaked in culture media (pH = 7.2–7.4). During the next odd days (0, 1, 3, 5, 7, 9), the hydrogel was separated from the fluid and was dried for 12 h to get the weight stabilized. The samples were lyophilized and final mass (Mf) were weighed and rate of weight loss was estimated based on the following equation:

Wt% = 100 × (Wt −Wo)/WO, where, WO is the initial dry weight and Wt is the dry weight of the samples at time. The degradation rate was analyzed based on change in hydrogel weight before and after incubation in media during the days aforementioned and finally the weight loss was figured out.

### 1–4 Mouse preantral follicles isolation and encapsulation

14-day-old NMRI (Naval Medical Research Institute) mice derived from the animal house of Royan institute (Tehran, Iran) were housed in conditioned environment (12 h light, 12 h dark). Each animal was sacrificed with cervical dislocation and the ovaries were removed from the body and placed in 50μm droplet of alpha Minimal Essential Medium (α-MEM; α-MEM; Gibco; Paisley, UK) with 10% fetal bovine serum (FBS (Gibco. USA)). The preantral follicles (100 to 150 μm in diameter) were mechanically isolated using 29-G needle. After collecting all preantral follicles, they were evaluated for viability, using 20 μl of 0.4% trypan blue for one second and then pipetted five times into α-MEM medium. Follicles were immediately observed under invert stereomicroscope (Nikon, Tokyo, Japan) and the ones stained blue were considered dead and eliminated, while the non-colored follicles were used for encapsulation and randomly divided and allocated to both experiments of follicular 3D in vitro culture and in vivo transplantation.

The preliminary evaluation of WJH, ALG and WJH+ALG droplets revealed that WJH lacked the adequate solidity for encapsulation of ovarian follicles and could not be hold by syringe (Fig 1G); therefore, the study was further proceeded using ALG and WJH+ALG for encapsulation and transplantation of preantral follicles. Isolated preantral follicles were collected from α-MEM medium and inserted inside 5-μl droplets of ALG or WJH+ALG using pulled glass pipette. It is worth mentioning that all the follicles isolated from each individual mouse were encapsulated into a single gel droplet and the number of isolated follicles ranged from 9 to 16 per mouse. Further, gel droplets were placed in the calcium bath for 60 seconds for alginate polymerization. Next, the scaffolds were observed under invert stereomicroscope in order to ensure that the ovarian follicles were successfully encapsulated in ALG and WJH+ALG scaffolds. Finally, the grafts, which were further used for transplantation, were merely composed of one of encapsulated auto-preantral follicles.

### Step 2: 3D in vitro culture (IVC) of mouse preantral follicles

**Fig 2:**
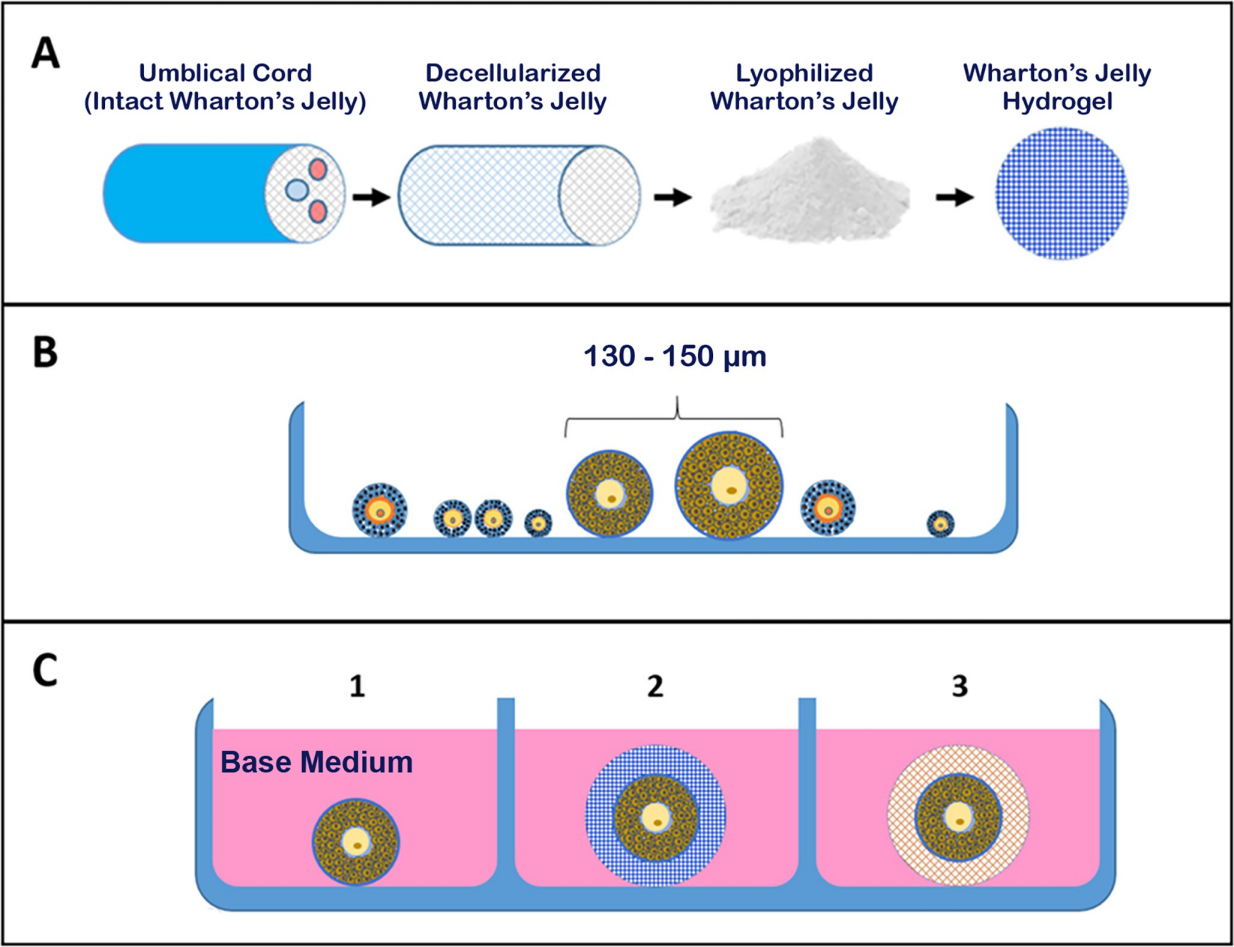
Different stages of follicular isolation and 2D and 3D culture. A) Preparation of Wharton’s jelly hydrogel, B) Follicular isolation and C) 3D in vitro follicular culture using HWJ (C-2) and ALG (C-3). All experiments were compared with 2D (C-1) culture of the follicles in base medium.

7 replicates were performed in the in vitro culture of follicles. After follicular isolation, pre-antral follicles diameter about 130–150 μm were allocated and shared in the 3 groups. Mouse isolated preantral follicles were shared between 3 in vitro culture groups and individually cultured in 96-well plates with 100μl α-MEM containing 10% FBS, 1% ITS and 100 mIU FSH, at 37°C and 5% CO_2_, for 13 days (base medium). Considering this rule that 40 μL of culture medium was exchanged with new medium every 2 days. The study groups were categorized as follow (Fig 2):

**Group 1 (2D group)**: preantral follicles were individually cultured in base medium.

**Group 2 (ALG group)**: preantral follicles were individually encapsulated in Alginate 0.75% and cultured in base medium.

**Group 3 (WJH+ALG group)**: preantral follicles were individually encapsulated in hydrogel composite of Wharton’s jelly hydrogel+alginate and cultured in base medium.

### Step 3: 3D in vivo transplantation of mouse preantral follicles

The site of transplantation was preliminarily validated through using whole ovary transplantation [14] Subsequently, the main experiment was conducted, in which preantral follicles were isolated from ovaries and then randomly divided in 3 groups:

1. **Intact ovary group**: 5 times for whole intact ovaries were transplanted in subserosal facia region.
2. **ALG group**: Isolated preantral follicles were encapsulated in ALG and transplanted in subserosal facia region in 5 times.
3. **WJH+ALG group**: Isolated preantral follicles were encapsulated in WJH+ALG composite and transplanted in subserosal facia region in 7 times.

Finally, the grafts retrieved after two weeks of transplantation and then evaluated.

### 3–1 Evaluation of transplantation site

**Fig 3:**
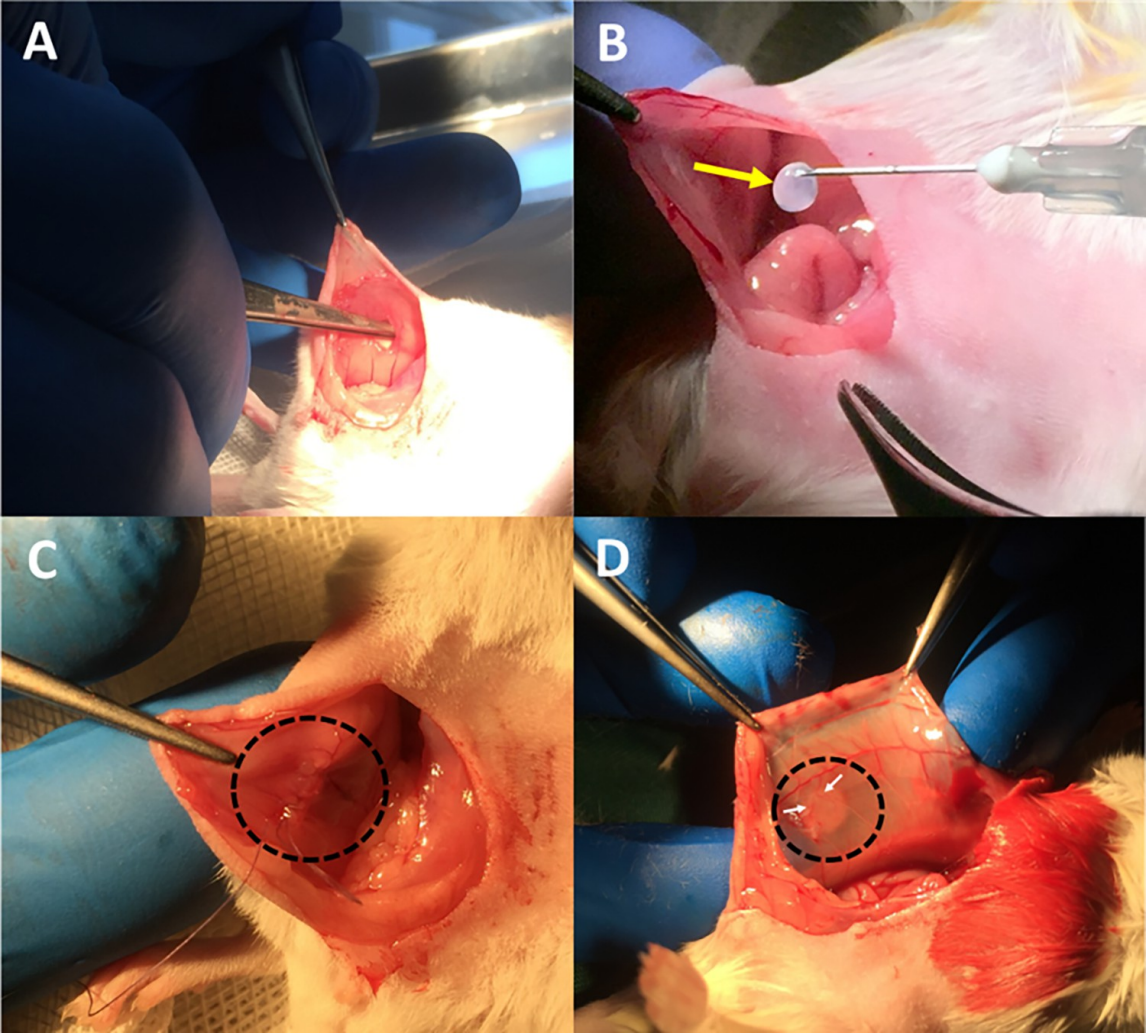
A) Incision of subserosal fascia to create pouch for insertion of ovarian tissue or follicular transplant. B) Grafting of encapsulated follicles in the site of transplantation. Yellow arrow denotes prepared bead for transplantation. C) Suturing of subserosal fascia at the site of transplantation (black dashed circle). D) Retrieval of follicular graft two weeks after transplantation with a black dashed circle showing the scaffold. White arrow indicates the blood vessels in vicinity of transplant.

Mice (n = 5) received intraperitoneal injection of ketamine (100 mg/kg; Alfasan, Woerden, the Netherlands) and Xylazine (10 mg/kg; Alfasan, Woerden, the Netherlands) cocktail to induce general anesthesia and a midline incision was performed for further oophorectomy. Then, upon making a small incision in the subserosal fascia, their intact ovary was inserted inside the cavity between subserosal fascia and muscular layer of abdominal wall (Fig 3A). Finally, the incisions in peritoneal wall as well as midline at separate layers were sutured (Fig 3C). The mice were kept in a clean warmed cage for recovery after surgery. In order to prevent any postoperative infections, they were treated with topical antibiotic ointment (Tetracycline-Naji 3.0%, Irannaji Co., Tehran, Iran) for three days after surgery. Following the two-week transplantation, the artificial ovary was retrieved and fixed in 4% formalin for histological and immunohistochemical analyses.

### 3–2 Auto-transplantation of artificial ovary in peritoneum

The mice were anesthetized and prepared using the same method described for evaluation of transplantation site. The scaffolds were placed inside the pouch between subserosal fascia and muscular layer of abdominal wall (one graft per each mouse; Fig 3B), which was created by incising subserosal fascia (Fig 3A). Thereafter, the peritoneal wall and midline were sutured (Fig 3C), and mice were kept in a clean warmed cage until recovery from anesthesia. The mice were treated with topical antibiotic ointment for three days after surgery. Transplanted scaffolds were retrieved two weeks later for histological examination, and to avoid loss of grafted tissue, a portion of surrounding tissue was excised as well (Fig 3D).

#### 3-2-1 Histological examination

Tissues and transplants were fixed using 4% formalin, processed by automated tissue processor and embedded in paraffin for histological analysis. Further, the samples were sectioned with 5 μm thickness and stained by either hematoxylin and eosin (H&E) or immunohistochemistry technique with vascular endothelial growth factor (VEGF) and CD34 as factors promoting angiogenesis following transplantation. Finally, slides were assessed using optic microscope (Nikon, Tokyo, Japan).

#### 3-2-2 Immunohistochemistry (IHC) assay

**Fig 4:**
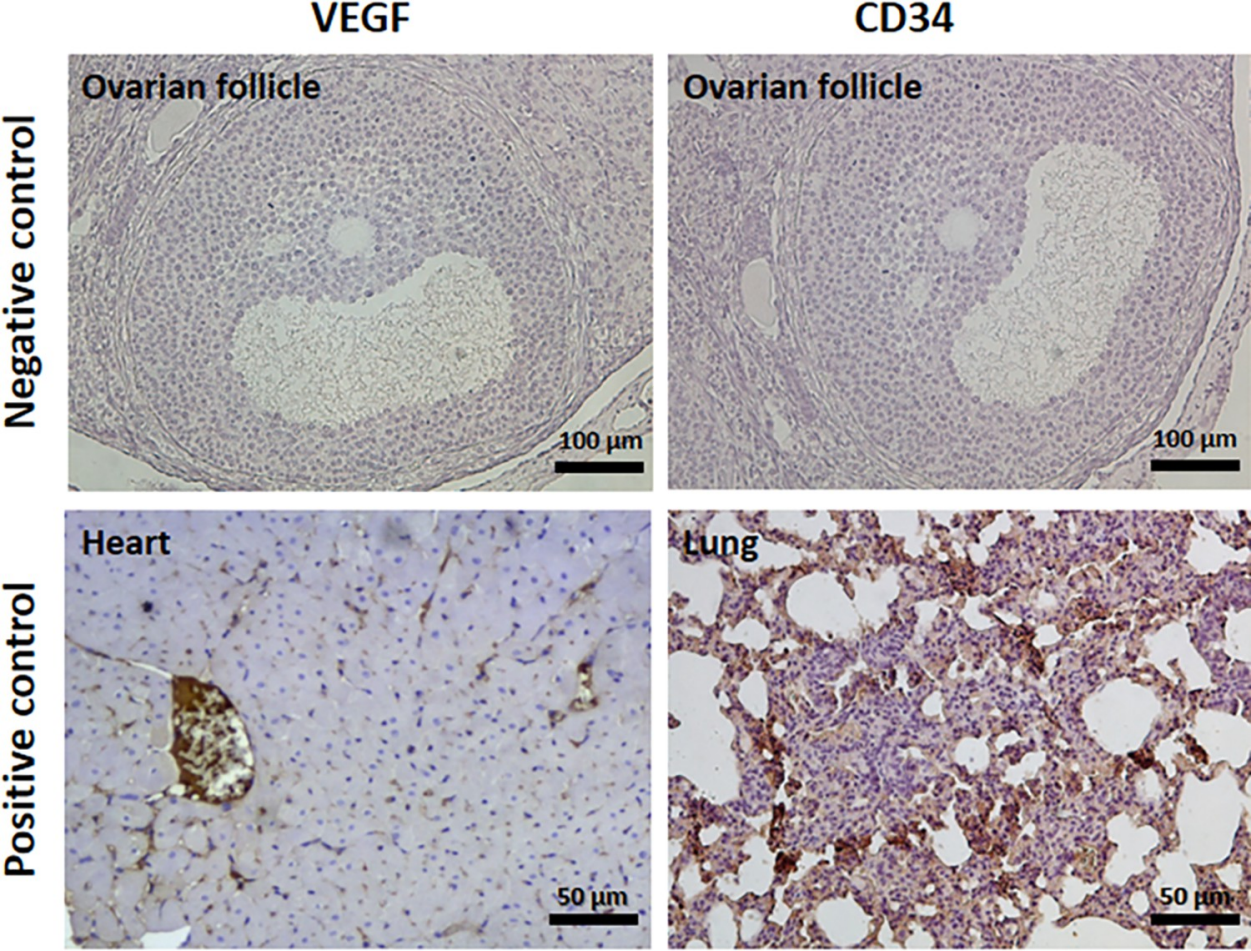
Negative and positive controls for immunostaining of VEGF and CD34. For preparation of negative control, the histological slides of ovarian follicles were merely treated with primary antibody and DAB, but not secondary antibody. Heart and lung tissues were used as positive controls of VEGF and CD34, respectively.

Initially, slides were deparaffinized using xylene and rehydrated by graded alcohol. Next, they were incubated with TBS at 95°C for 15 min to retrieve antigens and then washed with PBS. Slides were treated with hydrogen peroxide (H_2_O_2_) for 30 min at RT to block endogenous peroxidase activity and washed by PBS afterwards. Thereafter, they were treated with primary antibodies of VEGF (Santa Cruz Biotechnology Inc. TX, USA) and CD34 (Abcam, Cambridge, UK) at the dilution of 1:100 and incubated at 4°C overnight. After washing with PBS, slides were treated with secondary antibody for 1 h at room temperature, which was followed with washing by PBS and treatment with diaminobenzidine (DAB) solution (Diagnostic BioSystems, CA, USA). Finally, the slides were counterstained with hematoxylin. Negative controls were subjected to the same protocol excluding the primary antibody (Fig 4). For positive internal control, tissue of heart and lung were used for VEGF and CD34, respectively, based on manufacture’s indications (Fig 4). Quantification of VEGF and CD34 staining was implemented using ImageJ software (National Institutes of Health, Bethesda, MD, USA) and the corresponding data are presented as intensity staining proportion.

### 3–3 Statistical analysis

Initially, data were tested for normal distribution using Kolmogorov-Smirnov test (UNIVARIATE procedure) in order to log-transform data without normal distribution before analysis. Thereafter, data were analyzed using t-test (comparisons with two groups) or GLM (comparisons with more than two groups) procedures. In case required, LSMEANS statement was used for multiple comparisons. All analyses were conducted in SAS (SAS Institute Inc., Cary, NC, USA). Data are presented as mean ± SEM. Differences with P<0.05 were considered significant.

## Results

### Histological analysis of intact and decellularized Wharton’s jelly

**Fig 5:**
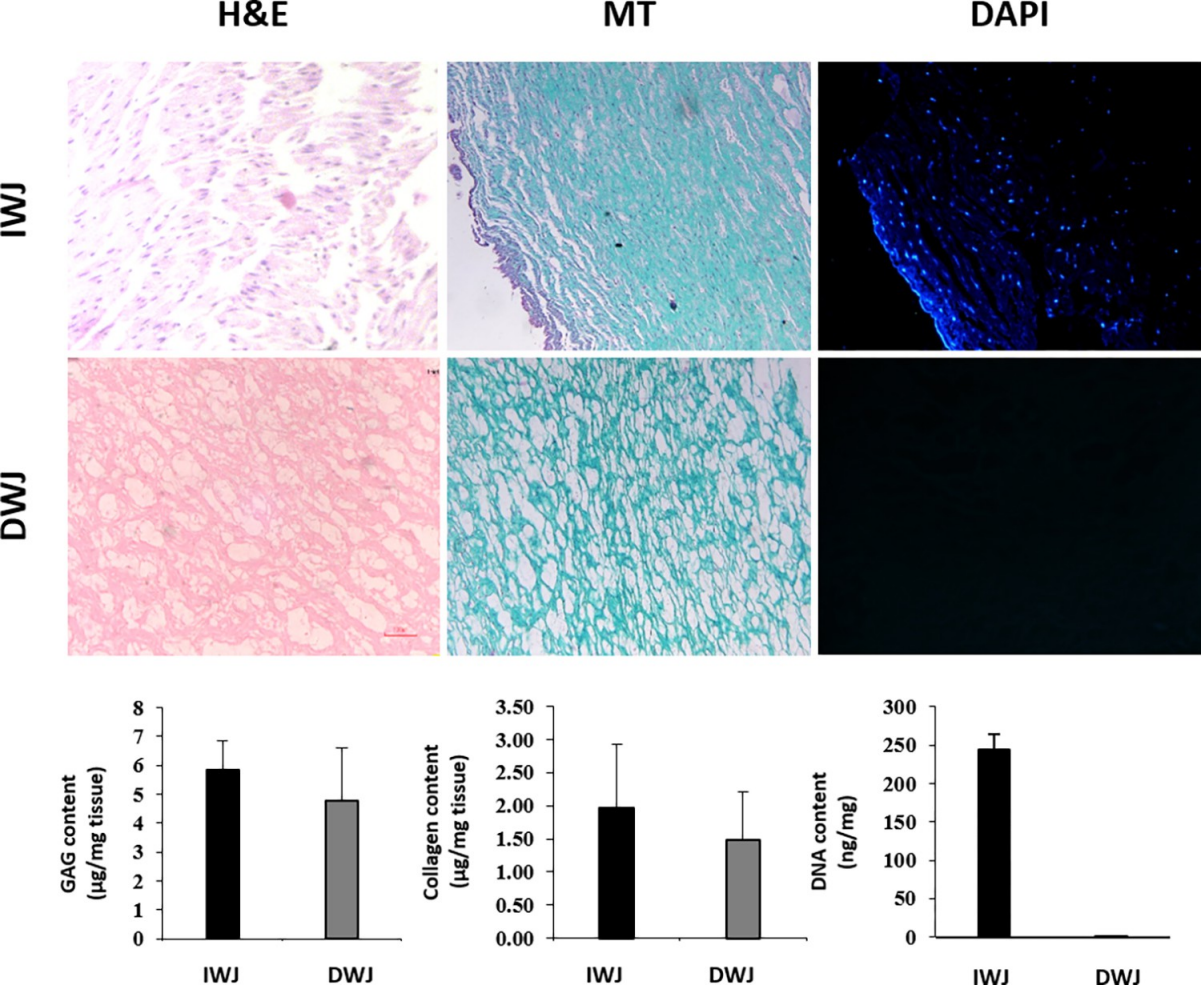
Characterization of intact and decellularized human WJ. Histological analysis for confirming the absence of cell nuclei and demonstrating the preservation of DWJ structure compared with IWJ. Data are presented as mean ± SEM. MT: Mason Trichrome, IWJ: intact Wharton’s jelly, DWJ: decellularized Wharton’s jelly.

Due to assess the impact of decellularization process on ECM structure, the histological analyses were performed. The cell nuclei remnants could not be tracked in ECM coming along with multiple various staining methods H&E and DAPI and it was found out that decellularization process was successful in eliminating the cells (Fig 5). Besides, the Masson’s Trichrome (MT) staining demonstrated that Collagen fibers was well preserved after decellularization process (Fig 5).

### DNA content, Collagen and GAG quantification quantities in decellularized Wharton’s jelly

The quantity of extracted DNA was checked on aforementioned protocol and the data from intact and decellularized tissues show that the mean content of intact WJ was 244.00 ± 19.73 per ng/mg while in DWJ this rate was 1.33± 0.15 ng/ mg. This data indicates that the majority of DNA remnants were removed in the matrix of WJ. The Collagen content in intact and decellularized WJ revealed that the decellularization procedure had no significant effect on Collagen content compared to the control (P>0.05) (1.49±0.72). In addition, the data from GAG assay showed that the decellularization treatment had no significant impact on the control group (P>0.05) (4.77±1.82) (Fig 5).

### Ultrastructure of human Wharton’s jelly

**Fig 6:**
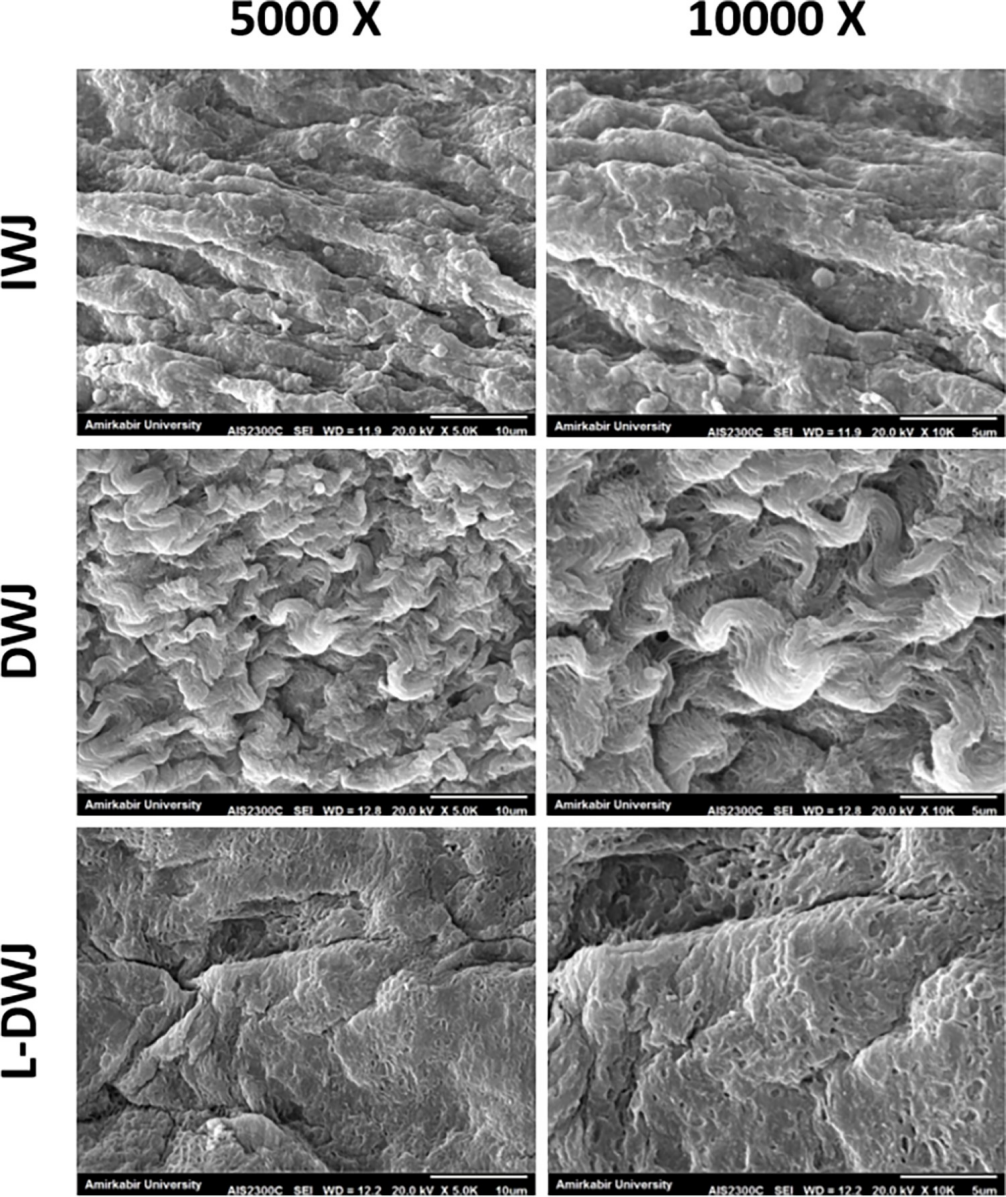
Scanning Electron Microscopy (SEM) from intact Wharton`s jelly (IWJ), decellularized Wharton`s jelly (DWJ), lyophilized decellularized Wharton`s jelly (L-DWJ).

Prior to lyophilization process, the particles were found to be evenly distributed in the polymer matrix which leads to forming these porous substrates. The morphological characteristics of the HWJ were evaluated before and after decellularization procedure. Our results confirmed that, the decellularized tissues were devoid of the cells. Further, it showed the good maintenance of WJ ECM 3D structure in decellularization and also the architecture of ECM was remained intact (Fig 6).

### Rheological assessments Wharton’s jelly hydrogel and alginate

**Fig 7:**
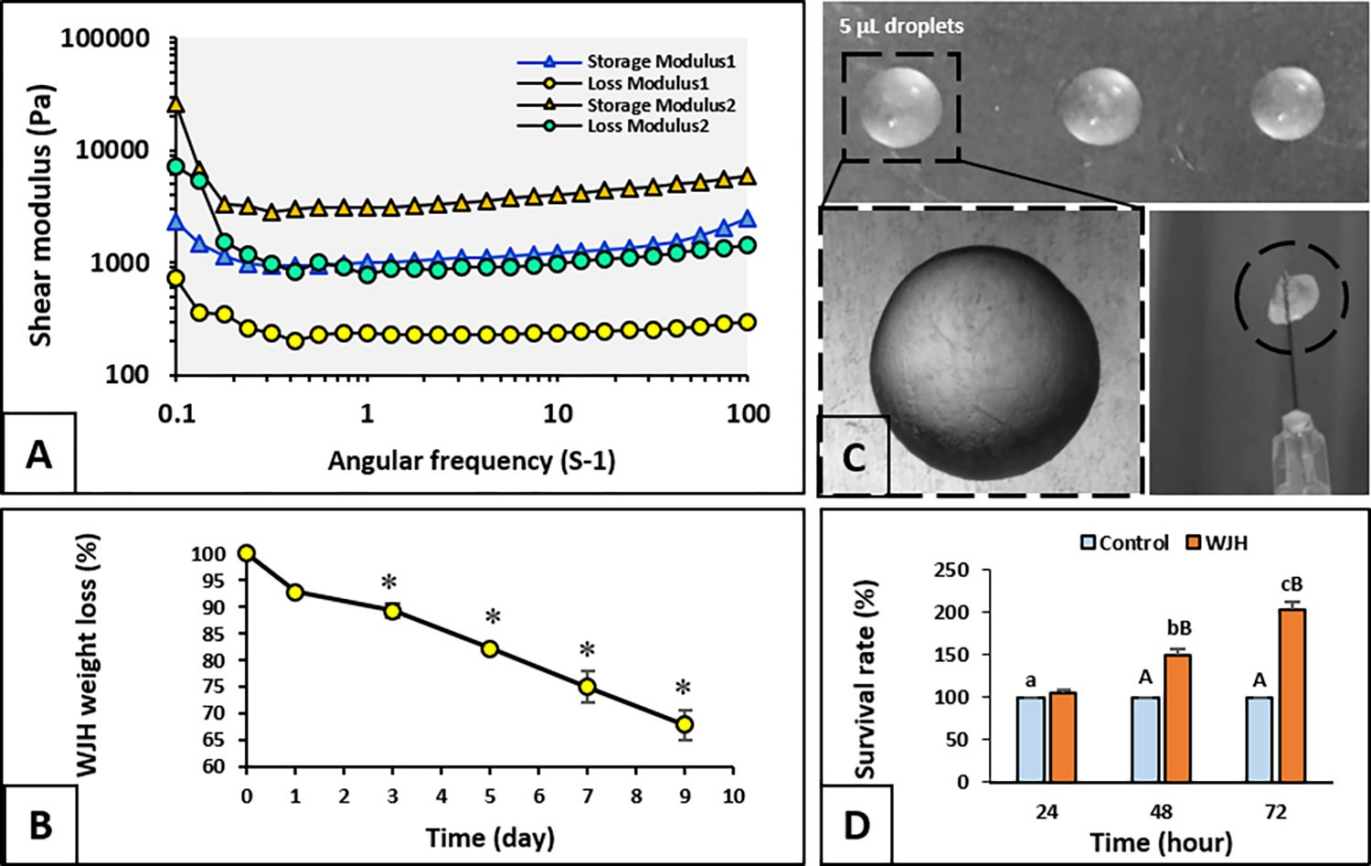
Rheological assessment of Alginate and Wharton’s Jelly hydrogel. A) Shear modulus of WJH (Wharton’s jelly hydrogel). B) Pattern of WJH (Wharton’s jelly hydrogel) degradation over a 9-day period. Asterisk (*) indicates significant difference in comparison to the original weight of WJH (P < 0.05). Data are presented as mean ± SEM. C) Droplets of WJH (dashed circle: hardened Wharton’s jelly hydrogel), D) Survival rate during MTT assay in the control and WJH groups. Small letters (a, b and c) indicate significant difference within a group over time (P < 0.05). Capital letters (A and B) indicate significant difference between two experimental groups on the specified time point (P < 0.05).

The rheology investigation showed the modulus G’ & G" as viscoelastic fluid properties of WJH+ALG and ALG hydrogels. The results revealed this fact that in both hydrogels storage modulus (G’) has got a remarkable difference as compared with the loss modulus (G") in which represents to an appropriate gelation after crosslinking in both hydrogels. Also, the ratio of G’ to G" (Gelation factor) in ALG is higher than WJH+ALG composition (Fig 7A).

### Cell metabolic activities in presence of WJH

Survival rate of MEFs did not change over time in control group. Yet, it was higher on/during 48 h than 24 h, and on/during 72 h compared with 24 and 48 h (P<0.001). In hour 24, survival rate did not differ between the control and WJH groups; however, on/during hour 48 and 72, it was higher in WJH than the control group (P<0.001) (Fig 7).

### WJH degradation over time and evaluation of the weight loss

The hydrogel weight loss was assessed; coming to the degradation rate of WJH showed a significant weight loss from day 3 onward (P<0.001) and reached 67.86 ± 2.88% of its original weight on day 9 of experiment (Fig 7).

### In vitro 3D culture of ovarian follicles: morphology and antrum formation

**Fig 8:**
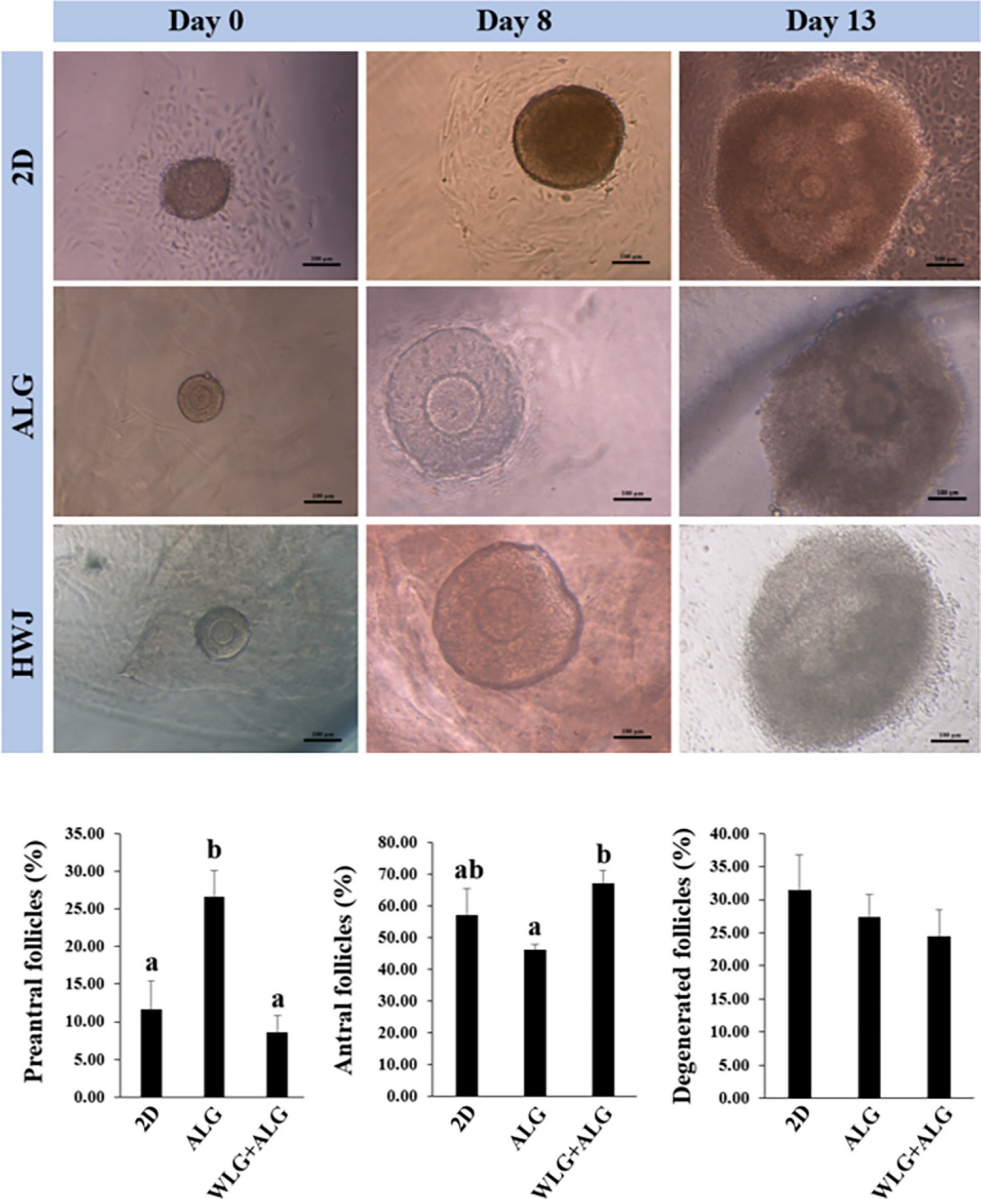
Development and survival of preantral and antral follicles following in vitro culture in 2D, alginate (ALG) and WJH+ALG groups. Various letters (a with b) indicate significant difference between groups (P < 0.05).

Preantral follicles with diameter of 130–150 μm were isolated from the ovary and divided under a randomized distribution condition in ALG, WJH and 2D for in vitro culture purpose. The cultured follicles within ALG and WJH matrices kept their spherical architecture, and clearly covered with granulose cells. Further, disorganization morphologies along with scattered granulosa cells were found in mouse ovarian preantral follicles cultured in two-dimensional system (Fig 8). In order to find out the impact of WJH and ALG on the follicular growth, the antrum formation volume of follicles was evaluated at the end of culture period (13 days). We realized from the data that the proportion of arrested preantral follicles was higher in ALG than 2D and WJH groups (P<0.05); however, it did not differ between 2D and WJH groups (P>0.05). The ratio of antral follicles in WJH group was greater than ALG group (P<0.05), but there was no difference among the other groups (P>0.05). Proportion of degenerated follicles did not differ among various experimental groups (P>0.05) (Fig 8).

### Evaluation of transplantation site

At the time of retrieval, the grafted scaffold was easily identifiable and seemed shrunk compared with its size at the time of grafting (Fig 3D). Furthermore, evident vasculature was macroscopically observed at the site of transplantation (Fig 3D).

**Fig 9:**
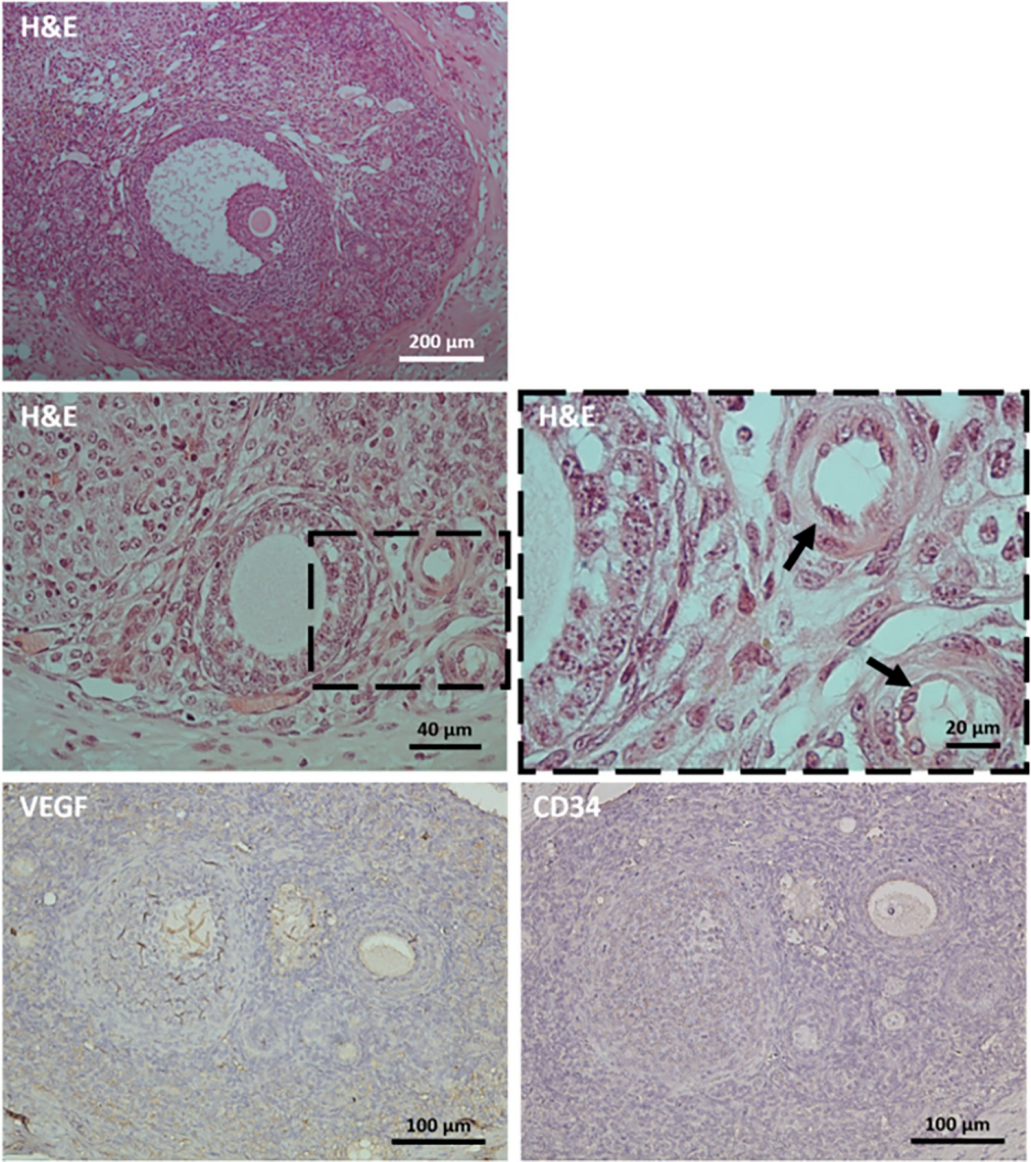
Successful development of ovarian follicles and vasculature as well as expression of VEGF and CD34 in whole ovary transplanted to the cavity between subserosal fascia and muscular layer of abdominal wall. Dashed areas delineate site of detected vasculature and black arrows denote blood vessels.

Histological examination showed development of follicles in whole ovaries transplanted to the cavity between subserosal fascia and muscular layer of abdominal wall (Fig 9). Additionally, development of vessels throughout grafted ovaries, including the periphery of ovarian follicles, was observed (Fig 9). Moreover, immunohistochemistry assay indicated expression of VEGF and CD34 all over the transplanted ovaries (Fig 9).

### Microscopic features of the transplanted follicles in ALG and WJH+ALG groups

**Fig 10:**
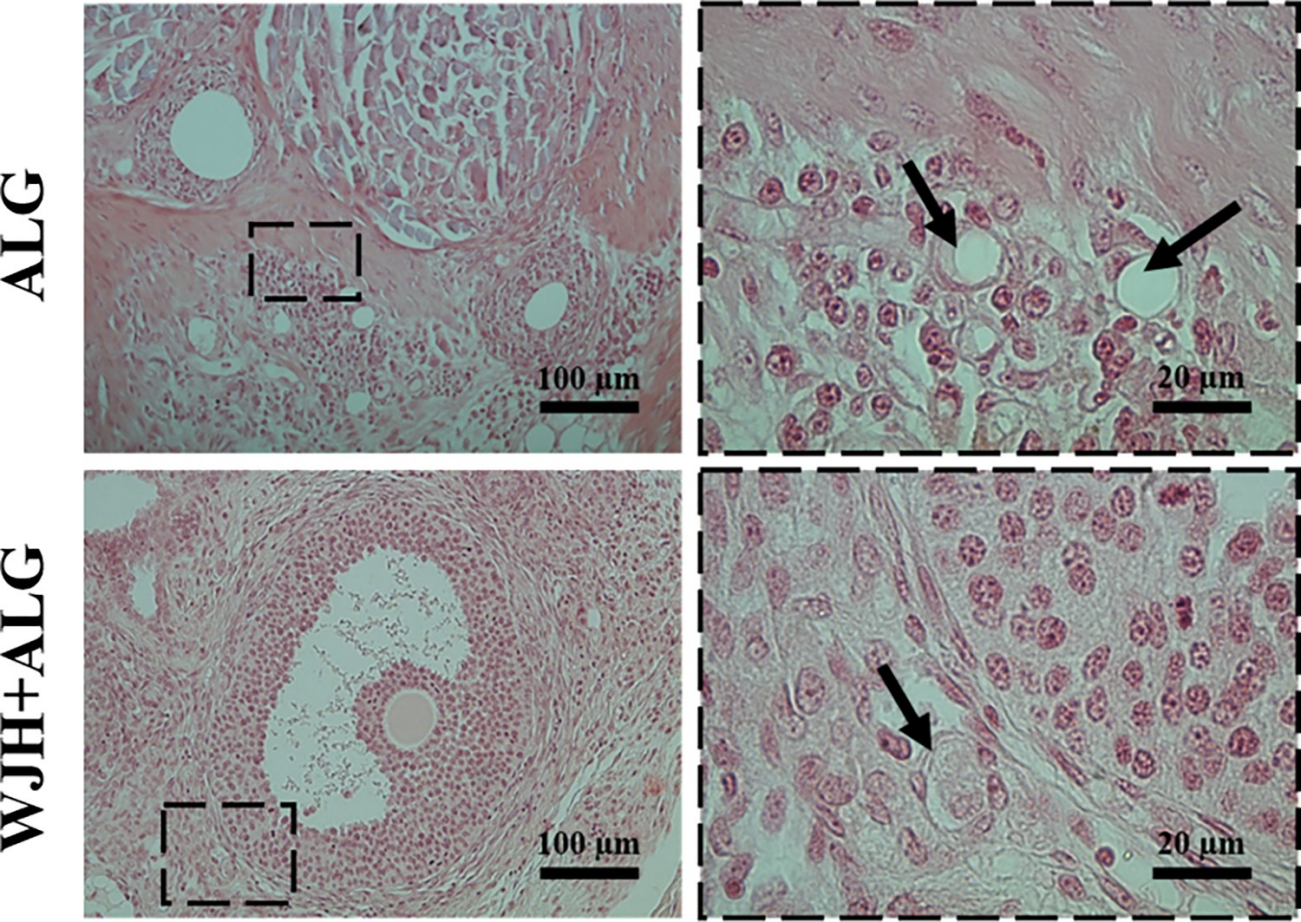
Cellular infiltration and development of vasculature in grafted scaffolds in ALG and WJH+ALG groups. Dashed areas delineate site of detected vasculature and black arrows denote blood vessels.

In the retrieved transplants, stromal cells were observed in the matrix of both ALG and WJH+ALG groups. Given that the grafts were merely consisted of scaffolds (ALG or WJH+ALG) and preantral follicles, observation of these cells implicate cellular infiltration from surrounding tissues during post-transplantation period (Fig 10). Moreover, development of vasculature was also noticed in both ALG and WJH+ALG groups, and in WJH+ALG group, blood vessels were detectable in the periphery of follicles (Fig 10).

**Fig 11:**
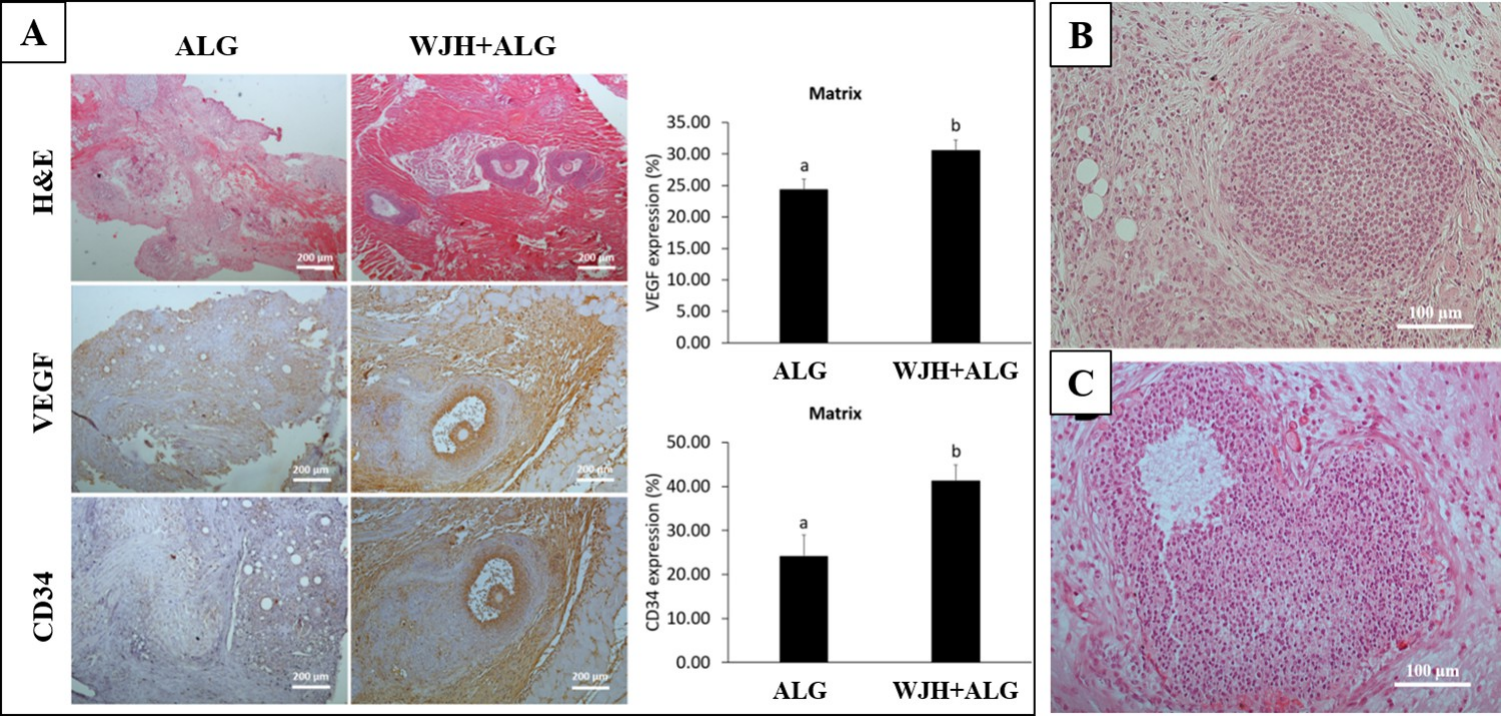
Development of transplanted follicles to antral stage and expression of VEGF and CD34 in WJH+ALG versus ALG group (A). Various morphologies of ruptured follicles observed in WJH+ALG group (B, C). Data are presented as mean ± SEM. Various letters (a with b) indicate significant difference (P < 0.05).

Further, no preantral follicles were observed in ALG and WJH+ALG groups (Table 1 and Fig 11A). Also, no antral or ruptured follicle was present in ALG group. However, the development of follicles up to antral stage and remnant of ruptured follicles was evident in WJH+ALG group (P<0.05; Table 1 and Fig 11A). Survival of encapsulated preantral follicles either as antral or cellular mass remaining after follicular rupture was higher in WJH+ALG than ALG group (P<0.05; Table 1).

**Table 1:** Development of encapsulated preantral follicles following transplantation in ALG and ALG+WJH groups.

	Experimental group	
Parameter	ALG (n = 5)	ALG+WJH (n = 7)
No. of encapsulated follicles per transplant	12.00 ± 1.00	12.86 ± 0.91
No. of preantral follicles	0.00 ± 0.00	0.00 ± 0.00
No. of antral follicles	0.00 ± 0.00^a^	1.86 ± 0.34^b^
No. of ovulated follicles	0.00 ± 0.00^a^	1.43 ± 0.30^b^
Proportion of follicles that developed to antral stage (%)*	0.00 ± 0.00^a^	25.04 ± 3.74^b^
Ovulation rate (%)^§^	0.00 ± 0.00^a^	40.24 ± 8.22^b^

*Proportion of follicles that developed to antral stage was calculated by dividing the number antral and ovulated follicles detected in each transplant by the number of encapsulated follicles. ^§^Ovulation rate was calculated by dividing number of ovulated follicles by total number of follicles observed in each transplant. ^a, b^ Values with different superscripts within rows differ (P < 0.05).

In histological examination, there were some structures with various morphologies similar to a mass of granulosa cells and devoid of antrum, which were probably the remnant of antral follicles that were ruptured inside the graft (Fig 11B and 11C). Some of the remnants of antral follicles had consistent spherical structure (Fig 11B), but others were vacuolated, which was probably resulted from the incomplete evacuation of follicular fluid (Fig 11C).

### Microscopic comparison of WJH+ALG transplant versus intact ovary

**Fig 12:**
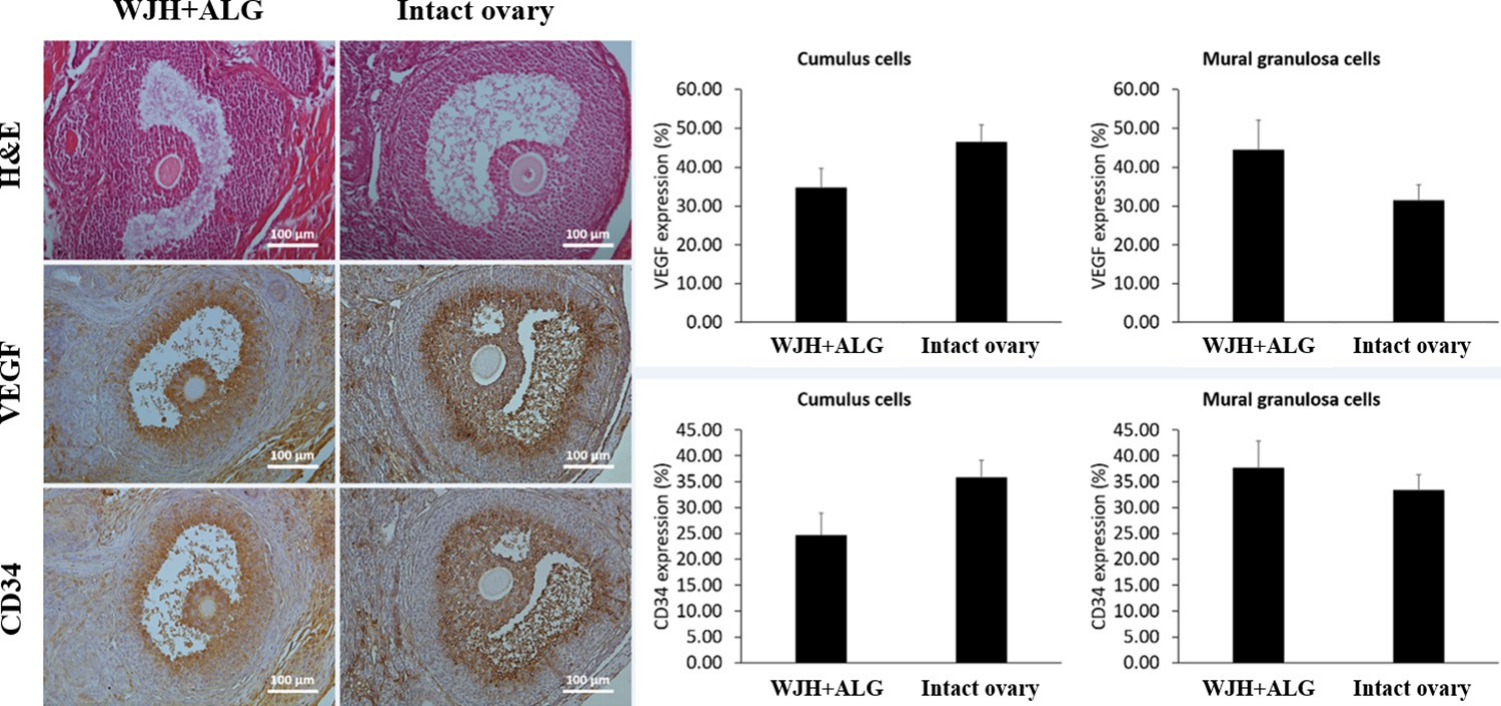
Morphology as well as pattern and level of VEGF and CD34 expression in antral follicles developed in WJH+ALG scaffolds as compared with antral follicles of intact ovaries. Data are presented as mean ± SEM.

**Fig 13:**
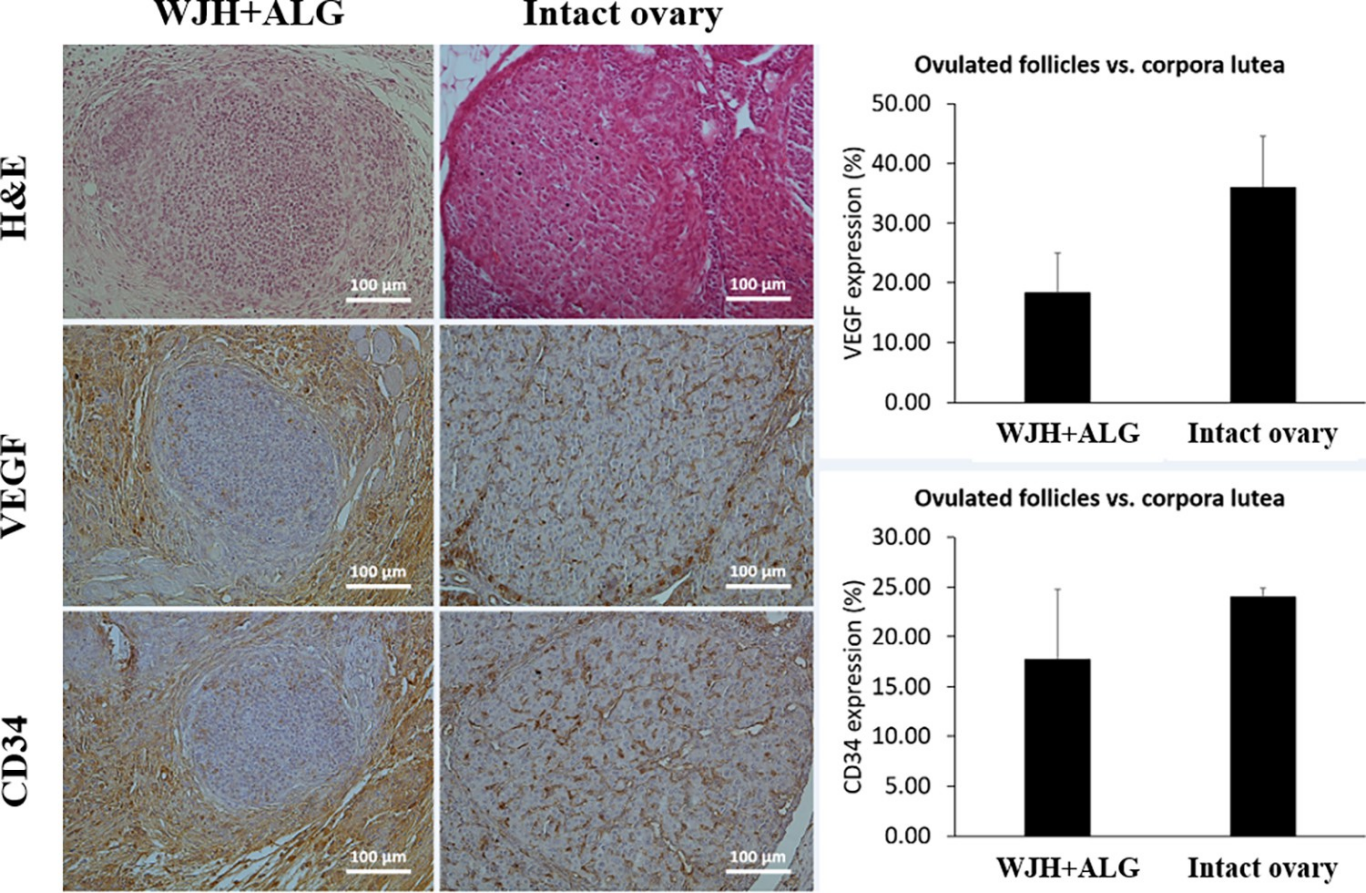
Morphology and expression of VEGF and CD34 in ovulated follicles in WJH+ALG group compared with corpus luteum in intact ovaries. Data are presented as mean ± SEM.

Antral follicles developed inside WJH+ALG were comparable to antral follicles detected in intact ovaries in terms of morphology (Fig 12). In spite of rupture in a proportion of antral follicles in WJH+ALG group, the resultant structure did not resemble the putative corpus luteum. Indeed, the size of cells in remnants of collapsed follicles was smaller than luteal cells composing corpus luteum in intact ovaries; moreover, the cellular cord organization, which was observed in corpus luteum of intact ovaries, had not formed in remnants of ruptured follicles inside WJH+ALG transplants (Fig 13).

### Pattern of angiogenic factors (VEGF and CD34) expression

Expression of both VEGF and CD34 in mural granulosa cells were observed inside ALG and WJH+ALG transplanted ovaries, in which it was higher in WJH+ALG group compared with the ALG group (P<0.05; Fig 11A).

In cumulus cells, intensity of CD34 and VEGF expression did not differ between antral follicles developed following encapsulation with WJH+ALG and antral follicles of intact ovaries (Fig 12). Additionally, the pattern of VEGF and CD34 expression was comparable between antral follicles of transplants and intact ovaries in mural granulosa cells, and in this regard, the expression of both VEGF and CD34 was more pronounced in the cells of outer layer interfacing antrum as compared with the deep cells (Fig 12).

Although collapsed follicles did not form corpora lutea, the expression of VEGF and CD34 in the remnants of collapsed follicles in transplants was not different from that in corpora luteal belonging to intact ovaries (P> 0.05; Fig 13).

## Discussion

To preserve the fertility of women and young girls suffering from cancer or other reproductive disorders, in vitro culture of ovarian follicles [15] and transplantation of ovarian tissue can provide an important link from bench to clinic [16]. The initial challenge to expand the follicular culture is to define proper biomaterials for supporting ex vivo growth of follicles [17,18]. In order to create a link between tissue engineering and reproduction, some different matrices have been constructed to promote follicular maturation and subsequently produce meiotically competent oocytes to develop novel assisted reproductive techniques for preservation of fertility [19,20]. Encapsulation of the ovarian follicles in the matrix maintains the whole architecture of follicles [21]. Although there is a wide range of different synthetic and natural scaffolds accessible for tissue engineering, some of natural polymers including collagen, gelatin, silk, chitosan and elastin have been proved to have issues in terms of processing, purity and protein denaturation. Considering the synthetic materials, the alloys derived from metals seem tough to deal with and resistant to biodegrading [22,23]. Polymers such as poly lactic-acid (PLLA), Polyglycolic acid (PGA), Polycaprolactone (PCL), Poly lactic acid-co-glycolic acid (PLGA) have got suitable properties to be applied for constructing an appropriate scaffold; however, they are basically synthetic, and hence poor in biological features [24]. Therefore, there is a growing demand on developing a suitable natural scaffold material. ECM- based is a fruitful material including; growth factors, fibronectin, and various types of collagens in which cells can be attached, proliferated, differentiated and hence is used as a natural scaffold for in vivo and in vitro procedure. Taking the advantages and disadvantages of all natural and synthetic scaffolds, we have reached to this opinion that DWJ has got the most appropriate characteristics as an ECM. WJ is one of those natural scaffolds that is used due to its capability as growth factor secretion, biocompatibility and biodegradable, low immunogenicity, availability, anti-inflammatory, ethical acceptability, easy bio-banking, low preparation cost [25]. Wharton’s Jelly consists of various proteins including collagen, laminin, elastin and a wide range of growth factors which play a role in cell growth, proliferation, adhesion and differentiation. Decellularized WJ matrix thoroughly exposed appropriate tissue engineering characteristics/properties. While performing the decellularization process, it was found that the bioactivity of the WJ matrix was enhanced in which a considerable amount of angiogenic VEGF as well as a passive release of macromolecules set free. The main core of this article, which is conducted for the first time, falls on the WJ used as natural hydrogel to improve the outcome of preantral mouse follicles culture and then transplantation [26]. There are many methods published in some studies for decellularization of WJ such as chemical, physical and enzymatically treatments [4,26]. Utilizing the chemical treatments including sodium dodecyl sulfate (SDS) and triton X-100, is not fruitful due to declining the level of growth factors and also the constriction of the fibers, such as GAGs and collagen which may be damaged through the process [27,28]. To avoid this, we picked up suitable method to decellularized WJ so that it leaves less negative impact on ECM components. Some data from the Wilshaw et al. represented that SDS did not leave a negative impact on collagen content of human amniotic membrane (HAM) [29]. Besides, Haghshenas et al. revealed that after decellularization of HAM with SDS, there is no significant decrease in collagen content [30]. In the study of Amensag and McFetridge and also Motamed et al. showed that SDS plays a helpful role to eliminate the cells from amniotic membrane [31]. Also, in decellularization of the heart [32], kidneys [33], skin [34], tendons [35], the SDS has been applied in a wide range.

To seize our goals in this research study, the decellularization procedure was performed based on SDS, Tris and EDTA. Applying EDTA, as an alone component to decellularization, is ineffective and should be in conjunction with enzymatic methods. Histological analysis, H&E and DAPI staining showed that the majority of nuclear and cellular materials were removed after decellularization process and the DNA quantification confirmed this result. Collagen is momentous in the tissue engineering because plays an important role in cell proliferation and adhesion and hence its perseverance is so critical after decellularization. Quantitative data from the GAG and collagen indicate that the ECM is preserved after decellularization procedure in DWJ. Some prior research studies have applied natural ECM resources from human and animal, such as liver, contained great amount of collagens (types III, IV, V, and I), hyaluronic acid, and several sulfated GAGs. This fact not to be neglected in this study that decellularization process also had no significant effects on ECM content. It is approved by MTS technique that in vitro viability and proliferation of MEFs through supplementation of WJ conditioned medium in culture medium was nontoxic for the cells applied in the medium.

Following the first phase of the present study, we evaluated the positive impact of the WJH on mouse preantral stage follicles during the 14 days of 3D culture. Wang et al., 2014, applied the basic fibroblast growth factor (bFGF) in single follicular 3D culture in the alginate bead, as supportive factor during 13-day culture. He showed that the diameter and survival rate remarkably goes up in comparison with the other groups without bFGF [36,37]. It illustrated the presence of various growth factors especially bFGF supports the human follicular growth.

Improving the effects of hydrogel systems and also supporting in vitro culture of ovarian follicles in several hydrogel-combinations have been proposed up to now [38]. Low degradation time of ALG prevents the follicular development in comparison with the other scaffolds and hydrogels that support follicular development by providing a dynamic mechanical environment [39]. According to the report published by Eun Jung Kim et al., applying the combination of hyaluronan and hydrogel supports follicle growth, estradiol secretion and meiotic resumption in oocytes. Mendez et al., have set up a polyethylene glycol-hydrogel combination system through which the in vivo physiological state gets fit into in vitro [38]. Kim et al., 2020, figured out the impact of extracellular matrix-derived soft hydrogel (ES-hydrogel) on in vitro follicle 3D culture in comparison with alginate which is widely used in the 3D culture of ovarian follicles [40]. The data suggested that ES-hydrogel had short and thin units of filaments whereas the alginate had very dense ECM structure. Further, ES-hydrogel supports folliculogenesis more impressively than alginate in vitro 3D culture. In our current research, the highest antral follicular formation rate (near to 70%) showed an important privilege of WJH+ALG composition compared to alginate. The project conducted by Hassani and her colleagues showed that chitosan as a permissive hydrogel can support growth and integrity of follicles during in vitro culture [41]. Also, Nikniaz et al.,2021, compared various protocols to decellularize of ovarian tissue and following that, measured the follicular survival rate in extracellular matrix (ECM)-alginate scaffold. Whole decellularization procedures were impressive to drive cells from ovarian cortex, while the residual DNA content in SDS-Triton-Ammonium group was very low and the cytobiocompatibility for follicle was higher than the other groups and is an optimal option for in vitro follicular culture [42]. In addition, Agar is found the same appropriate and effective as Matrigel-coated inserts to be applied for the survival and growth of follicles during culture, and consequently it can be considered as an inexpensive alternative substrate to culture frozen-thawed human ovarian cortical strips performed in Ghezelayagh et al., research project [43]. Recently, Jamalzaei and her colleagues, evaluated the impact of hyaluronic acid-alginate (HAA) hydrogel integrated with ovarian cells (OCs) on culturing of the ovarian mouse follicles [44]. The data showed that HAA hydrogel is an encouraging hydrogel for mouse ovarian follicular in vitro culture. In addition, the results showed that applying OCs improves the follicular culture in consideration to hydrogel type.

Wharton’s Jelly is an ECM rich in collagen, hyaluronic acid (HA) and glycosaminoglycans and also contains various bioactive molecules such as peptide growth factors, principally insulin like growth factor-1 (IGF-1) and transforming growth factor-β (TGF-β) in which are appropriate factors for follicular growth and development in vitro. Besides, WJ is a rich source of reservoir of mesenchymal stromal cells which has been applied for various clinical particle with proven reparative effects following skin graft procedure and treatment of foot ulcers and diabetic ulcers with osteomyelitis [45,46].

The present study revealed that the composite of alginate with WJH could successfully support development of preantral follicles toward antral stage as well as expression of VEGF and CD34 in the transplant. However, alginate did not contribute to survival and development of ovarian follicles and resulted in less pronounced expression of such angiogenic factors. By contrast, Vanacker et al. [47] showed that encapsulation of preantral follicles with alginate matrix could assist not only cell proliferation but also the survival and development of follicles with follicle recovery rate of 20%. One possible explanation for such discrepancy could be the higher number of preantral follicles encapsulated in alginate droplet in the study conducted by Vanacker et al. indicated that vascularization was not consistent all over the transplant and majorly localized to its periphery, probably due to the slow degradation rate of alginate. Therefore, improvement in survival and development of encapsulated follicles in WJH + ALG group could partly be attributed to WJH imparting the scaffold greater level of degradability, which would facilitate follicular growth. It is also worth noting that unlike alginate, WJ contains a myriad of extracellular matrix proteins [48,49] which could have potentially favored construction of a more familiar microenvironment for grafted follicles. In addition to the biochemical cues provided by the WJ bioactive components, follicle development can be facilitated by the biophysically conditioned hydrogel of WJH+ALG which may recapitulate the native microenvironment of the ovarian tissue. In this regard, our mechanical assessment by rheometry showed that by addition WJ into the ALG hydrogel, the storage modulus as an indication of matrix stiffness was tuned to more closely mimic the stiffness of native tissue [30]. Taken together, failure of ALG to support development of ovarian follicles in this study could not implicate that ALG alone may not be used for construction of artificial ovary since numerous previous studies have been substantiated the usefulness of ALG for this purpose [50–52] and the results of the present study in this regard were probably originated from its special conditions, including limited number of encapsulated follicles in each graft due to autotransplantation model, absence of ovarian cells alongside with transplanted follicles and the physical and chemical characteristics of ALG used in this study. Besides, WJH alone could not be used for construction of beads for transplantation as the WJH droplets were not solid enough to be handled during the process of transplantation (Fig 1). However, addition of WJ into the matrix of ALG, allowed for sufficient gelation of ALG as G’ was still higher than G” with a plateau trend at subsequent frequencies in rheological assessments which revealed an acceptable mechanical stability of WJH+ALG beads for easy handling during the culture and transplantation. Indeed, the composite of ALG and WJH was not only solid enough for construction of transplantable beads but could successfully support development of grafted preantral follicles in the autotransplantation model applied in this study. Hence, growth factor properties of WJH might have also assisted in the elevated level of angiogenic factors expression in the grafts as well as the successful development of preantral follicles to antral stage.

Interestingly, the pattern of angiogenic factors expression was comparable between antral follicles formed post-transplantation and native antral follicles. The similar pattern of angiogenic factors expression may implicate normal development of transplanted follicles in the grafts. Moreover, approximately 40% of the survived follicles in the WJH+ ALG group were devoid of antrum, which could have resulted from follicular rupture. Yet whether this rupture was associated with ovulation was not assessed in the present study. Given the results of the present study, it appears that the composite of alginate with WJH could support rupture of antral follicles but failed to trigger morphological development of corpus luteum. Regardless, the main intention for transplantation of encapsulated preantral follicles is to enhance their development up to antral stage, so that the engulfed oocyte could be retrieved from the graft and be used for ensuing reproductive purposes. Irrespective of scaffold material, the present study also substantiated that subserosal fascia of abdominal wall could serve as a functional transplantation site for either ovarian tissue or encapsulated follicles, which is in accord with the findings of the study conducted by Tavana et al. [14] in rat.

## Conclusion

In conclusion, the present study showed that the composite of alginate and WJH could support in vitro and in vivo development of encapsulated murine preantral follicles up to the antral stage. Different studies revealed that WJ hydrogel as a rich source of ECM components can improve in vitro condition and provide an appropriate environment for culture of isolated ovarian follicles. In addition, WJH enhanced the expression of VEGF and CD34 as contributing factors to angiogenesis in transplanted encapsulated follicles. Yet further research is required to evaluate the application of this scaffold for foresight purposes in human fertility preservation.
